# Characteristics of young people with long term conditions close to transfer to adult health services

**DOI:** 10.1186/s12913-015-1095-6

**Published:** 2015-09-30

**Authors:** Hannah Merrick, Helen McConachie, Ann Le Couteur, Kay Mann, Jeremy R. Parr, Mark S. Pearce, Allan Colver, on behalf of the Transition Collaborative Group

**Affiliations:** Institute of Health and Society, Newcastle University, Sir James Spence Institute, Royal Victoria Infirmary, Newcastle upon Tyne, NE1 4LP UK; Institute of Neuroscience, Newcastle University, Sir James Spence Institute, Royal Victoria Infirmary, Newcastle upon Tyne, NE1 4LP UK

**Keywords:** Transition, Young people, Complex health needs, Long term conditions, Participation, Wellbeing, Satisfaction with services

## Abstract

**Background:**

For many young people with long term conditions (LTC), transferring from paediatric to adult health services can be difficult and outcomes are often reported to be poor. We report the characteristics and representativeness of three groups of young people with LTCs as they approach transfer to adult services: those with autism spectrum disorder with additional mental health problems (ASD); cerebral palsy (CP); or diabetes.

**Methods:**

Young people aged 14 years-18 years 11 months with ASD, or those with diabetes were identified from children’s services and those with CP from population databases. Questionnaires, completed by the young person and a parent, included the ‘Mind the Gap’ Scale, the Rotterdam Transition Profile, and the Warwick and Edinburgh Mental Wellbeing Scale.

**Results:**

Three hundred seventy four young people joined the study; 118 with ASD, 106 with CP, and 150 with diabetes. Participants had a significant (*p* < 0.001) but not substantial difference in socio-economic status (less deprived) compared to those who declined to take part or did not respond. Condition-specific severity of participants was similar to that of population data.

Satisfaction with services was good as the ‘gap’ scores (the difference between their ideal and current care) reported by parents and young people were small. Parents’ satisfaction was significantly lower than their children’s (*p* < 0.001). On every domain of the Rotterdam Transition Profile, except for education and employment, significant differences were found between the three groups. A larger proportion of young people with diabetes were in a more independent phase of participation than those with ASD or CP. The wellbeing scores of those with diabetes (median = 53, IQR: 47–58) and CP (median = 53, IQR: 48–60) were similar, and significantly higher than for those with ASD (median = 47, IQR: 41–52; *p* < 0.001).

**Conclusions:**

Having established that our sample of young people with one of three LTCs recruited close to transfer to adult services was representative, we have described aspects of their satisfaction with services, participation and wellbeing, noting similarities and differences by LTC. This information about levels of current functioning is important for subsequent evaluation of the impact of service features on the health and wellbeing of young people with LTCs following transfer from child services to adult services.

## Background

Young people with long term conditions (LTC) often find transfer from children’s services to adult services difficult [1, 2]. A number of reports have identified the need to improve transfer [3–5] but implementation has been slow [6–8]. There is a small amount of literature on the effectiveness of transitional care programmes [9], which includes the Diabetes Navigator study [10], joint clinics for kidney transplant patients [11], transition coordinators [12], and the LIFEspan model [13] but these studies were disease or setting specific.

The need to transfer from child-centred to adult oriented healthcare systems is part of the wider set of tasks that young people with LTCs need to negotiate. In this context ‘transition’ is a wider concept than transfer and defined as ‘the purposeful, planned process that addresses the medical, psychosocial, educational and vocational needs of adolescents and young adults with chronic medical and physical conditions’ [14]. Understanding transition as a process that should address these wider needs of young people has focused attention on understanding the wellbeing and participation of young people with LTC as they move to adulthood. Several LTCs are associated with reduced participation in employment and education, independent living and social participation [15–18]. However it is not known to what extent such poor outcomes were already present before transfer to adult services because there is a lack of longitudinal research exploring the experiences of young people before, during and after healthcare transfer [9].

The Transition Research Programme [19] aims to promote the quality of life and health of young people with LTCs by generating evidence to enable National Health Service (NHS) Commissioners and Providers of healthcare in the UK to facilitate successful transition of young people from child to adult health care, thereby improving health and social outcomes. One component of the Programme is a longitudinal study that aims to identify the features of healthcare that are effective and efficient, and to examine how these features contribute to positive outcomes for young people, including wellbeing, participation and satisfaction with services. While there is some preliminary evidence of the benefit of transition programmes in diabetes care [7, 20], there has been little research with young people with complex physical impairments [21]. Further, the lack of planned transfer to adult mental health services for young people with neurodevelopmental disorders has been highlighted [6]. For these reasons, a cohort of young people with one of three LTCs was recruited: individuals with autism spectrum disorder (ASD) who continued to access services for additional mental health problems, as an exemplar of neurodevelopmental disorder; cerebral palsy, as an exemplar of complex physical impairment; and diabetes as an exemplar of chronic illness.

These three exemplar conditions, ASD with additional mental health problems, cerebral palsy and diabetes, were deliberately chosen because it seemed probable that the groups would have some similar and some distinct needs with respect to their negotiation of transfer to adult services. A young person with ASD may have difficulty in interpreting advice given and in understanding the need for specialist help for persisting or recurring mental health problems. Young people with cerebral palsy may have multiple impairments such as visual or communication difficulties as well as motor problems; and are likely to need to attend a number of different specialist clinics. A young person with a chronic illness, such as diabetes, is likely to experience episodes of poor health and will usually require lifelong medication.

This paper describes satisfaction with services, participation and wellbeing in the three groups of young people with LTCs. While transition pathways have been established in much diabetes care there has been little progress in developing services for those with chronic physical impairment or neurodevelopmental disorders [22]. Therefore, we hypothesized that young people and parents with diabetes would report higher satisfaction with services than those with cerebral palsy or ASD. We also hypothesized that the young people with cerebral palsy and those with ASD would report lower levels of independence in life activities than those with diabetes [15–17]. Finally, because mental health problems are associated with lower wellbeing scores [23], we hypothesized that the young people with diabetes or cerebral palsy would report higher wellbeing scores than the young people with ASD who also had additional mental health problems.

We also describe the sample for this longitudinal study and its representativeness in terms of: i) whether participants differed significantly from non-participants (those approached who declined to take part or did not respond) and ii) how the severity of the condition in our sample compared to that in population data for each of the three exemplar conditions.

## Methods

The study received a favourable ethical opinion from Newcastle and North Tyneside1 Research Ethics Committee (12/NE/0059). The study methods and measures used have been described in detail elsewhere [24] and are summarised below.

### Sample

Between June 2012 and October 2013 young people with diabetes and young people with ASD with additional mental health problems (*e.g.* ADHD, depression, Obsessive Compulsive Disorder, anxiety) were recruited from children’s services in five and four UK healthcare provider Trusts respectively. Young people with cerebral palsy were recruited from two regional population registers, the North of England Collaborative Cerebral Palsy Survey (NECCPS) [25] and the Northern Ireland Cerebral Palsy Register (NICPR) [26]. All participants were aged 14 to 18 years 11 months at recruitment and had not started the transfer of their healthcare to adult services. The young people had no significant learning disability, as assessed by the referring clinicians, and all could self-report. A parent or carer for each young person was also invited to take part in the study to complete some questionnaires.

### Procedures

Young people with ASD and young people with diabetes were approached about the research by their clinician. Young people with cerebral palsy were sent a letter inviting them to take part in the research after their clinician had confirmed they were eligible to take part. Information on date approached, date of birth, gender and postcode was collected for all young people who were approached. Postcodes were used in England to calculate the index of multiple deprivation (IMD) [27] for the English participants and non-participants, and in Northern Ireland to calculate the multiple deprivation measure (MDM) [28]. IMD and MDM are markers of community-level socio-economic status (SES); a higher IMD/MDM score indicates more socio-economic deprivation.

A locally-based research assistant (RA) visited each young person and their parent or carer at a venue to suit the participant, usually their home. The RA obtained informed consent from both the young person and their parent or carer and then administered the questionnaires. The young people completed questionnaires on their own, only helped by the RA if needed.

### Measures

Parents and young people completed respectively the 27 and 22 item ‘Mind the Gap’ scale [29] about their experiences of services, rating items on a Likert scale of 1–7. Satisfaction with services is conceptualised as the ‘gap’ score between ratings of best and current care. A positive ‘gap’ score indicates that ideal care is rated higher than current care, and the greater the ‘gap’ score the lower the level of satisfaction. Young people also completed the Rotterdam Transition profile [30], a nine domain questionnaire on participation, defined as involvement in life situations [31]. On each domain participants select the statement that best describes their current situation. Each statement represents one of three phases of transition; phase 1- childhood/dependent on parents, phase 2- experimenting and orienting to the future, and phase 3- adulthood/independence. The Warwick and Edinburgh Mental Wellbeing Scale (WEMWBS) is a 14-item questionnaire, developed in the UK and valid in the age range 13 to 21 years, that captures young people’s mental wellbeing [32].

For each condition group, condition-specific measures were also completed. Parents of participants with ASD completed the Social Responsiveness Scale (SRS) [33] to confirm the autism characteristics. The parents and the participants with ASD each completed versions of the Strengths and Difficulties questionnaire (SDQ) [34], a measure of emotional and behaviour problems. The SDQ has been used in several studies of young people with ASD as a brief screening instrument for potential mental health problems [35, 36]. In the cerebral palsy group, a severity of impairment questionnaire was completed by the RA which included the Gross Motor Function Classification System (GMFCS) [37]. For the diabetes group we obtained data from medical records about HbA1c (a measure of blood sugar level control) averaged over the year before recruitment and age at diagnosis as a proxy for likelihood of complications and more difficult blood sugar control.

### Sources of comparative data

In order to examine representativeness of our sample of young people with ASD and additional mental health problems, we identified two cohorts of young people with ASD who did not have significant learning disability. We obtained means and standard deviations on self-reported SDQ from an Australian sample of adolescents aged 12–16 years (mean age = 13.8 years; 26 boys and three girls) with Asperger’s syndrome [38]; and we obtained from the Special Needs and Autism Project (SNAP) parent-reported SDQ and SRS scores for a sub-sample, aged approximately 16 years with an IQ above 70, of their population based cohort of young people with ASD [39, 40]. For cerebral palsy we extracted data from the NECCPS [25] and NICPR [26]. GMFCS was used as a measure of severity; this was as recorded at age 5 years because population data on GMFCS were not available on 14–18 year olds. At one cerebral palsy site, GMFCS was not available at age 5 years (ten children). For diabetes we obtained means and standard deviations for age at diagnosis and HbA1c from the 2011/12 National Paediatric Diabetes Audit for young people aged 14 – 18 years [41].

### Data coding and statistical analysis

All scales were calculated using the appropriate manuals. Scales were checked for normal distribution and where non-normality was found the appropriate non-parametric test was used. The extent to which significant differences existed between participants and non-participants (those approached who declined to take part or did not respond) was investigated for age, gender and SES using t-, chi-squared, and Mann Whitney tests respectively. Data on non-participants were unavailable from one of the diabetes sites.

When considering the representativeness of the study sample with respect to severity of condition, chi-squared tests were used for comparisons of GMFCS for the cerebral palsy group, while t-tests were used for comparisons of SRS total scores, parent reported and young person reported SDQ for the ASD group, and for age of diagnosis and HbA1c levels for the diabetes group.

An 80 % rule was used for responses with missing data on the ‘Mind the Gap’ scale as recommended by its developers [29]. Overall satisfaction was calculated for young people who answered sixteen or more of the questions, and parents who answered twenty one or more of the questions. Kruskal-Wallis tests were used to assess differences in satisfaction scores across conditions. Differences in satisfaction scores between young people and their parents were assessed using Wilcoxon sign-rank tests. For the WEMWBS questionnaire, when only one answer was missing, an average of the participant’s other answers was imputed as recommended in the user’s guide [42]. Differences in wellbeing scores across conditions were assessed using Kruskal-Wallis tests. No imputation was done for the Rotterdam Transition Profile where questions were unanswered. Chi-squared tests were used to assess differences in phase of transition across conditions. All test statistic p-values of less than 0.05 were considered significant and statistical analysis was undertaken in Stata, version 12 [43].

No further psychometrics were carried out on the ‘Mind the Gap’ scale and the Rotterdam Transition Profile because they have been validated and used with young people with a variety of LTCs [29, 30]. However, for the WEMWBS internal consistency was assessed using Cronbach’s alpha with 95 % confidence intervals (CI) to check it was an appropriate measure to use in all three groups, particularly the ASD group with additional mental health problems.

## Results

### Participants and non-participants

**Fig. 1:**
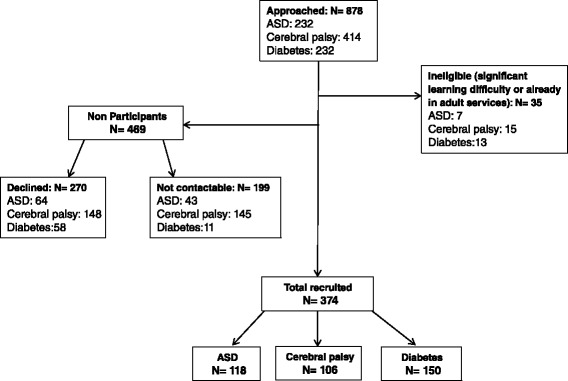
Flow diagram of recruitment. (ASD, Autism Spectrum Disorder)

Eight hundred seventy eight young people were approached and 374 were recruited to the study; 118 with ASD and additional mental health problems, 106 with cerebral palsy and 150 with diabetes (Fig. 1). For each young person a parent or carer was invited to participate; 369 agreed (367 parents, one grandparent and one foster parent). Thus, data from a parent or carer was available for 98.6 % of the young people.

Participants did not differ significantly from non-participants by age or gender (Table 1). Overall, participants had significantly (*p* < 0.001) lower SES scores (*i.e.* less deprived) than non-participants; however, the difference in overall IMD score on a continuous scale ranging from 0.5 to 87.8, was only 6.1.

**Table 1:** Summary statistics of participants and non-participants^a^

	Condition	Participants			Non-participants^b^			
Age		Mean	SD	n	Mean	SD	n	*p* value^1^
	ASD	16.1	1.3	118	16.0	1.3	99	0.68
	CP	16.5	1.9	106	16.5	1.4	293	0.80
	Diabetes	16.2	1.3	150	15.9	1.2	67	0.14
	Total	16.2	1.3	374	16.3	1.3	459	0.40
		Male	Female	Total	Male	Female	Total	
Gender		n (%)	n (%)	n (%)	n (%)	n (%)	n (%)	*p* value^2^
	ASD	82 (69)	36 (31)	118 (32)	81 (76)	25 (24)	106 (23)	0.24
	CP	60 (57)	46 (43)	106 (28)	181 (62)	112 (38)	293 (62)	0.35
	Diabetes	77 (51)	73 (49)	150 (40)	31 (45)	38 (55)	69 (15)	0.38
	Total	219 (59)	155 (41)	374 (100)	293 (63)	175 (37)	468 (100)	0.23
SES		Median	IQR	n	Median	IQR	n	*p* value^3^
IMD	ASD	19.9	9.7-31.1	118	22.6	15.9-37.8	96	0.01
	CP (NECCPS)	17.5	13.2-30.4	60	27.4	12.7-39.9	164	0.05
	Diabetes	12.1	7.3-20.8	150	14.3	10.3-23.2	67	0.11
	Total	15.5	8.6-28.0	328	21.6	12.2-36.8	327	<0.001
MDM	CP (NICPR)	15.3	10.7-26.8	46	17.7	8.4-34.8	128	0.58

^a^Young people approached who declined to take part or did not respond

^b^Data missing from one diabetes site

^1^t-test of a significant difference in means between participants and non-participants

^2^Chi-squared test of a difference in the gender distribution between participants and non-participants

^3^Mann-Whitney rank sum test of a difference in deprivation measure between participants and non-participants

*SD* Standard Deviation

*IQR* Interquartile Range

*SES* Socio-economic status as captured for England by index of multiple deprivation (IMD) [27] and for Northern Ireland by multiple deprivation measure (MDM) [28]

*ASD* Autism Spectrum Disorder

*CP* Cerebral Palsy

*NECCPS* North of England Collaborative Cerebral Palsy Survey

*NICPR* Northern Ireland Cerebral Palsy Register

Three hundred seventy three/Three hundred seventy four young people and 366/369 parents completed sufficient items of the ‘Mind the Gap’ scale for analysis. Three hundred seventy four young people completed the Rotterdam Transition Profile, however due to missing responses, only 373 completed the domains for ‘employment and education’ and ‘leisure activities’, 367 for ‘romantic relationships’ and 359 for ‘services and aids’. Three hundred seventy three/Three hundred seventy four young people completed the WEMWBS. One YP did not complete a sufficient number of items to score the WEMWBS and four young people (1 %) had a missing value imputed.

### Comparison of condition severity of participants with population data

There were no significant differences in mean SDQ scores between participants with ASD and those in the Australian community source (Table 2). The parent-reported SDQ scores in young people with ASD were significantly higher than parent-reported SDQ scores from the UK SNAP community sub-sample (*p* < 0.001); as were the SRS total scores (*p* < 0.001). The GMFCS levels of the participants with cerebral palsy were representative of those of the same age in the NECCPS and the NICPR (Table 2). Participants with diabetes were representative of UK young people of the same age for age at diagnosis and HbA1c (Table 2).

**Table 2:** Comparison between participants and population data of condition severity by health condition

AUTISM SPECTRUM DISORDER							
Questionnaire	Participants			Australian source			
Young person SDQ	n	Mean	SD	n	Mean	SD	*p* value^1^
Total score	116	17.6	6.1	29	18.4	5.6	0.47
Emotional symptoms	116	4.8	2.5	29	4.5	3.0	0.68
Conduct problems	116	3.0	2.2	29	2.8	1.7	0.47
Hyperactivity	116	5.5	2.4	29	5.9	2.0	0.39
Peer Problems	116	4.2	2.2	29	5.2	2.7	0.07
Prosocial	116	6.5	2.1	29	7.0	2.0	0.23
	Participants			SNAP source			
Parent-reported SDQ	n	Mean	SD	n	Mean	SD	*p* value^1^
Total score	113	22.8	5.9	105	13.5	7.2	<0.001
Emotional symptoms	113	6.3	2.4	105	2.9	2.4	<0.001
Conduct problems	113	3.6	2.5	104	1.7	1.6	<0.001
Hyperactivity	113	6.7	2.5	105	4.8	2.8	<0.001
Peer Problems	113	6.2	2.2	105	4.1	3.1	<0.001
Prosocial	113	5.1	2.3	104	6.0	2.4	0.01
	Participants			SNAP source			
SRS	n	Mean	SD	n	Mean	SD	*p* value^1^
Total score	114	117.2	29.7	71	90.3	27.3	<0.001
CEREBRAL PALSY							
Questionnaire	Participants^a^			Regional samples: NECCPS & NICPR^b^			
GMFCS at age 5 years	n (%)			n (%)			*p* value^2^
I	25 (26)			95 (26)			
II	42 (44)			159 (44)			
III	16 (17)			59 (16)			
IV	11 (11)			41 (11)			
V	2 (2)			8 (2)			
Total	96 (100)			362 (100)			0.99
DIABETES							
Indicator	Participants			UK national data^c^			*p* value^3^
	n	Mean	SD	n	Mean	SD	
HbA1c (mmol/mol)	150	72.5	18.6	11676	71.6	22.6	0.56
Age at diagnosis (years)	150	9.5	3.8	11676	9.0	3.8	0.10

^1^Two sample *t*-test of difference in means between participants and population data

*SD* Standard Deviation

*SDQ* Strength and Difficulties Questionnaire

*SRS* Social Responsiveness Scale

*SNAP* Special Needs and Autism Project

^a^Data missing from one site

^b^Those born in region, 1995–98, IQ > 50

^2^Chi-squared test of difference in GMFCS distribution between participants and population data

*GMFCS* Gross Motor Function Classification System

*NECCPR* North of England Collaborative Cerebral Palsy Survey

*NICPR* Northern Ireland Cerebral Palsy Register

^c^Data from UK National Paediatric Diabetes Audit, children aged 14–18 years [39]

^3^Two sample *t*-test of difference in means between participants and comparators

### Satisfaction with services

Young people’s and parents’ expectations of their ideal and current care are shown in Table 3. The median group score for ideal care for each subscale was equal to or above five. For the young people in all three groups, the highest scores for ideal care related to *healthcare personnel*. For parents the highest scores for ideal care related to *care processes*. For the young people’s and parents’ ratings of their current care the highest scores related to *healthcare personnel* for all three groups.

**Table 3:** ‘Mind the Gap’ scores of young people and parents by health condition

	Young people				Parents/carers			
	ASD	CP	Diabetes		ASD	CP	Diabetes	
	Median (IQR)	Median (IQR)	Median (IQR)		Median (IQR)	Median (IQR)	Median (IQR)	
Ideal care								
Physical environment	5.0 (4.4, 5.8)	5.2 (4.4, 6.0)	5.4 (4.6, 6.0)		6.0 (5.2, 6.5)	6.0 (5.0, 6.5)	5.8 (5.2, 6.5)	
Healthcare personnel	5.9 (5.3, 6.6)	5.9 (5.3, 6.5)	6.0 (5.5, 6.5)		6.8 (6.3, 6.9)	6.5 (6.1, 6.8)	6.6 (6.0, 6.9)	
Care processes	5.8 (4.8, 6.4)	5.8 (4.8, 6.6)	5.7 (4.6, 6.2)		7.0 (6.6, 7.0)	6.9 (6.4, 7.0)	6.6 (6.0, 6.9)	
Current care								
Physical environment	4.2 (3.4, 5.0)	4.0 (3.4, 5.0)	4.6 (3.8, 5.0)		3.8 (3.0, 4.8)	3.7 (2.7, 4.5)	4.2 (3.2, 5.0)	
Healthcare personnel	5.1 (4.3, 5.7)	5.0 (4.1, 5.8)	5.6 (4.7, 6.1)		5.5 (4.4, 6.6)	4.6 (3.6, 5.5)	5.6 (4.6, 6.2)	
Care processes	4.6 (3.4, 5.4)	4.4 (3.0, 5.6)	4.6 (4.0, 5.6)		4.6 (3.1, 5.8)	3.6 (2.4, 5.0)	5.1 (4.0, 5.9)	
‘Gap’ score				*p* value^1^				*p* value^1^
Physical environment	0.8 (−0.2, 1.8)	1.0 (−0.2, 2.0)	0.8 (0.0, 1.8)	0.79	1.8 (0.5, 3.0)	2.0 (0.7 3.2)	1.4 (0.3, 2.7)	0.14
Healthcare personnel	0.6 (0.0, 1.5)	0.4 (0.0, 1.4)	0.4(−0.2, 1.1)	0.12	0.6 (0.0, 2.1)	1.6 (0.7, 2.8)	0.8 (0.1, 1.6)	<0.001
Care processes	1.0 (0.0, 1.8)	1.0 (0.0, 2.8)	0.6 (0.0, 1.4)	0.06	1.9 (0.6, 3.3)	3.0 (1.1, 4.1)	1.1 (0.3, 2.1)	<0.001

^1^Kruskal-Wallis test of differences in gap scores across long term health condition

IQR Inter-quartile range

ASD Autism Spectrum Disorder

CP Cerebral Palsy

No significant differences in the young people’s ‘gap’ scores were found between the three groups on any of the three subscales: *physical environment*; *healthcare personnel*, and *care processes* (Table 3). However, for parents there was a significant association between health condition group and ‘gap’ scores on two of the subscales; parents of young people with ASD reported the lowest ‘gap’ scores (being more satisfied) on *healthcare personnel* (*p* < 0.001), while on *care processes* (*p* < 0.001), parents of young people with diabetes reported the lowest ‘gap’ scores.

For the 363 cases where both parent and young person had completed a questionnaire, parents of those in the cerebral palsy and diabetes groups reported significantly lower satisfaction with services than their children across all three subscales (*p* < 0.001; not shown in Table 3). For the ASD group, parents reported significantly lower satisfaction than their children on *physical environment* and *care processes* (*p* < 0.001; not shown in Table 3).

### Participation

**Fig. 2:**
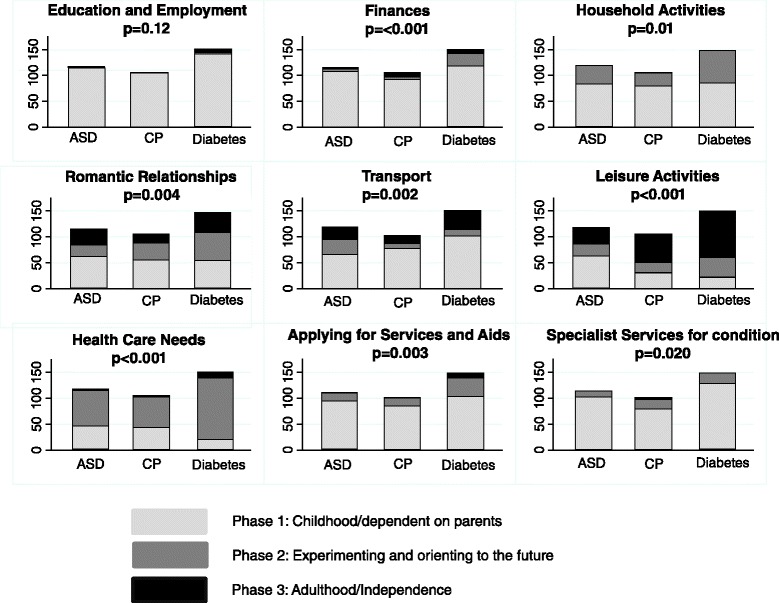
Numbers of young people in each phase of the Rotterdam Transition profile. (ASD, Autism Spectrum Disorder; CP, Cerebral Palsy)

On every domain of the Rotterdam Transition profile, with the exception of education and employment, there was a significant difference across the groups in the proportion of young people in each phase of transition (Fig. 2).

On six domains, larger proportions of the young people with diabetes were in a higher phase of transition than those with cerebral palsy: finances (*p* = 0.002), household activities (*p* = 0.005), romantic relationships (*p* = 0.03), leisure activities (*p* = 0.02), healthcare needs (*p* < 0.001), and applying for services and aids (*p* = 0.01). Similarly on seven domains, larger proportions of the young people with diabetes were in a higher phase of transition than those with ASD and additional mental health problems: finances (*p* = 0.01), household activities (*p* = 0.04), romantic relationships (*p* = 0.004), transportation (*p* = 0.003), leisure activities (*p* < 0.001), healthcare needs (*p* < 0.001), and applying for services and aids (*p* = 0.01). Finally on three domains, larger proportions of the young people with cerebral palsy were in a higher phase of transition than those with ASD and additional mental health problems: transport (*p* = 0.01), leisure activities (*p* < 0.001) and specialist services for my condition (*p* = 0.03).

### Wellbeing

**Fig. 3:**
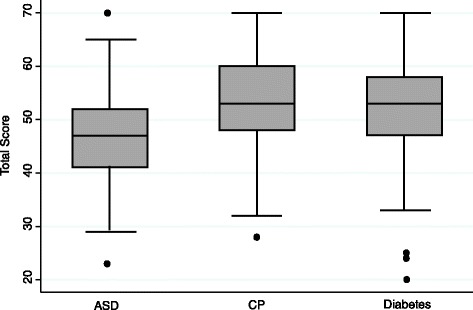
Box and whisker plots of wellbeing scores of young people by health condition. (ASD, Autism Spectrum Disorder; CP Cerebral Palsy. Boxes show median and interquartile range. Whiskers show adjacent values. Dots show outliers)

Cronbach’s alphas for the WEMWBS for those with ASD with additional mental health problems, those with cerebral palsy and those with diabetes showed good internal consistency (0.84, 95 % CI = 0.80-0.89; 0.87 95 % CI = 0.83-0.91; 0.88, 95 % CI = 0.88-0.93, respectively), supporting its suitability for use with all three groups. Figure 3 shows wellbeing scores for the three condition groups. As hypothesised, young people with cerebral palsy or diabetes reported significantly higher wellbeing scores (median = 53, IQR: 48–60, median = 53, IQR: 47–58; respectively) than those with ASD (median = 47, IQR: 41–52; *p* < 0.001, Kruskal-Wallis).

## Discussion

### Summary of findings and interpretation

This paper explores satisfaction with services, participation and wellbeing in three representative groups of young people with long term conditions who were close to transfer to adult services.

Our hypothesis that young people with diabetes would report higher satisfaction with services than those with cerebral palsy or ASD was not borne out. The gap between ratings of ideal and current care was small, which suggests that pre transfer, young people with different LTCs are generally happy with their interactions with services. However, significant differences were found on parents’ satisfaction with services; not only did parents’ satisfaction vary by health condition but also they were less satisfied than their children on all three subscales. This has been found in other studies [44–46] and indicates the importance of obtaining the perspective of both young people and their parents. There may be a range of explanations for why parents and young people have different expectations of healthcare services. For example it might be that parents have greater expectations of services, having enabled their child’s attendance at health clinics and other resources (indeed possibly fought to access services) over many years [29]. Or it might be that some parents find difficult the more young-person centred approach to health care during adolescence [47].

We had hypothesized that the young people with cerebral palsy or ASD would report lower levels of independence in life activities than those with diabetes. In each domain of the Rotterdam Transition Profile, except for education and employment, this was the case despite the groupings being of equivalent average age. For example, participants with ASD spent more leisure time on their own or at home with friends than the other two groups (ASD = 53 %, CP = 29 %, diabetes = 15 %). This is consistent with findings from the National Longitudinal Transition Study 2 that found young people with ASD were significantly more likely never to see friends outside of school and were socially isolated [15, 48]. However, there were also similarities; for example, many young people from all three groups (ASD = 85 %, CP = 85 %, diabetes = 69 %) were in phase 1 of the ‘applying for services and aids’ domain (still relying on their parents to apply for their services and aids). This reinforces the need for services for young people with LTCs to discuss with families, and actively promote, young people’s growing independence at the rate that is developmentally appropriate for each young person. It will be interesting to see how the patterns of autonomy across both the various life activities and the three exemplar conditions change over the three years of this longitudinal study, as young people and their parents negotiate the transfer of healthcare to adult services.

Finally, as hypothesized the young people with ASD and additional mental health problems reported lower wellbeing scores than the young people with diabetes or cerebral palsy. Further, the higher scores of those with CP or diabetes were similar to those of young people aged 16–25 years (median = 53, IQR: 42–62) in the Health Survey for England 2012 [23]. This is consistent with recent research showing young people with cerebral palsy have similar levels of wellbeing to those in the general population of the same age [49].

### Strengths and Limitations

We recruited 374 young people with LTCs to the longitudinal study. While recruitment rates were reasonably good for the ASD group (50.9 %) and diabetes group (64.7 %), the rate of recruitment of those with CP was lower (25.6 %). This may well be due to the method of recruitment which was to send letters of invitation, rather than for their clinician to approach young people and their families directly. Further, we only found small differences in socio-economic status between participants and non-participants.

A strength of this longitudinal study is that the results are likely to be generalisable to young people with a wide variety of LTCs for two reasons: first, the recruited young people have one of three very different LTCs; second, we have shown that our sample is representative of population data by condition-specific measures of severity.

The young people’s high questionnaire completion rates indicate that the instruments selected to measure the main outcomes of satisfaction with services, participation and wellbeing for the longitudinal study are acceptable to young people. The good internal consistency of the 14-items on the WEMWBS for each of the condition groups supports the suitability of this measure for all three groups.

A limitation was that we could not identify a population dataset containing SRS or SDQ scores for young people with ASD who were engaged with services due to an additional mental health problem. In our sample the parent reported SDQ and SRS scores for young people with ASD were higher than the equivalent scores from the SNAP sample collected at age 16 years but the SNAP sample was derived from a community study rather than being clinically referred. However, these higher scores confirm we were successful in obtaining a sample with complex health care needs rather than ASD alone, and thus likely to have continuing service needs as young adults [50]. A further limitation is that we could not compare our sample with the population data on age and socioeconomic status because these were not available.

## Conclusions

The health and social outcomes of young people with LTCs are often poor [3, 51–53]. However, it is not known to what extent the poor health status and levels of social functioning were present before transfer to adult services. We have successfully identified a large cohort of young people with LTCs and characterised aspects of their social functioning and satisfaction with healthcare services before the transfer of their healthcare to adult services. The results show the importance of noting the similarities between groups of young people with long term conditions as well as the differences. The young people and their parents have agreed to be contacted annually over the next three years. This will allow exploration of whether or not these baseline characteristics change through the transition period, and investigation of whether exposure to specific service features will impact on the outcomes defined for this study [24]. The period around transfer to adult health services coincides with development of adolescent identity, increasing awareness of healthcare and the need to balance it against the many other calls on a young person’s development and interests. Better understanding of what might constitute appropriate support for young people to enable them to engage successfully with adult healthcare services is much needed.

## Abbreviations

ASD: Autism spectrum disorder
CI: Confidence interval
CP: Cerebral palsy
GMFCS: Gross motor function classification system
IMD: Index of multiple deprivations
IQR: Interquartile range
LTC: Long term condition
MDM: Multiple deprivation measure
NECCPS: North of England Collaborative Cerebral Palsy Survey
NHS: National Health Service
NICPR: Northern Ireland Cerebral Palsy Register
RA: Research assistant
SDQ: Strengths and difficulties questionnaire
SES: Socio economic status
SNAP: Special needs autism project
SRS: Social responsiveness scale
WEMWBS: Warwick and Edinburgh Mental Wellbeing Scale
